# A recurrent *PAX6* mutation is associated with aniridia and congenital progressive cataract in a Chinese family

**Published:** 2012-02-16

**Authors:** Chongfei Jin, Qiwei Wang, Jinyu Li, Yanan Zhu, Xingchao Shentu, Ke Yao

**Affiliations:** Eye Center of the 2nd Affiliated Hospital, Medical College of Zhejiang University, Hangzhou, China

## Abstract

**Purpose:**

Aniridia is phenotyically and genetically heterogeneous. This study is to summarize the phenotypes and identify the genetic defect responsible for aniridia and congenital progressive cataract in a three generation Chinese family.

**Methods:**

A detailed family history and clinical data from patients were collected by ophthalmologic examination, including visual acuity, slit-lamp examination, tonometer, keratometry, corneal topography, optical coherence tomography, and ultrasonic A/B scan. All exons and flanking intronic sequences of the paired box 6 (*PAX6*) gene were amplified by polymerase chain reaction (PCR) and screened for mutation by direct DNA sequencing. Structure and function of the mutant PAX6 were analyzed by bioinformatics analysis.

**Results:**

All the six patients shared common manifestations of complete aniridia, congenital cataract and thickened cornea, and broad phenotypic variability was observed in nystagmus, ptosis, strabismus, glaucoma, corneal pannus, corneal curvature, corneal vascularization, cataract subtype, ectopia lentis, axial length, and optic disc anomalies. Sequencing of the candidate gene detected a heterozygous c.307C>T transition in the coding region of *PAX6*, resulting in the substitution of a highly conserved arginine codon for a termination codon (p.R103X). The p.P103X mutation co-segregated with the affected individuals in the family. The change was supposed to cause structural and functional changes based on computational analysis.

**Conclusions:**

We identified a recurrent *PAX6* c.307C>T mutation in an aniridia and congenital progressive cataract family, and summarized the variable phenotypes among the patients, which expanded the phenotypic spectrum of aniridia in a different ethnic background.

## Introduction

Aniridia (OMIM 106210) is a rare bilateral panocular disorder characterized by complete absence or partial absence of the iris [1]. It is associated with a range of other ocular abnormalities including aniridia-associated keratopathy (AAK), ectopia lentis, cataract, glaucoma, nystagmus, foveal hypoplasia, and optic nerve hypoplasia, which results in vision loss [2,3]. About two thirds of the cases of congenital aniridia are inherited as an autosomal dominant trait with high penetrance and variable expressivity [4,5]. The aniridia gene has been mapped on chromosome 11p13 by linkage analysis and positional cloning. The pair box gene 6 (*PAX6*) located at 11p13 is confirmed to be the major gene associated with aniridia [6-9].

Congenital cataract (OMIM 604307) is an opacification of the eye lens resulting in visual impairment or even blindness during infancy or early childhood [10]. According to the Human *PAX6* Allellic Variant Database [11], over 60 *PAX6* mutations have been reported to be associated with aniridia accompanied with congenital cataract. However, identified mutations are located throughout the length of *PAX6* with limited clear evidence of genotype-phenotype correlation.

In this study, we present the clinical and molecular genetic evaluations performed on a three generation aniridia and congenital progressive cataract family of Chinese origin.

## Methods

### Clinical data evaluation

**Figure 1:**
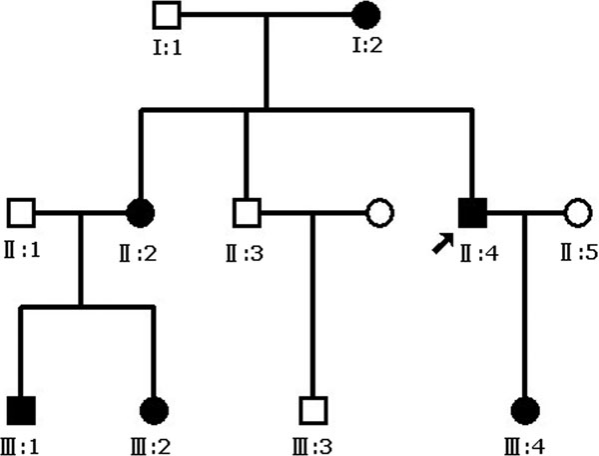
Pedigree of the aniridia and congenital progressive cataract. It is inherited as an autosomal dominant trait. The proband is marked with an arrow. Squares and circles indicate males and females respectively. Black and white symbols represent affected and unaffected individuals, respectively.

A family having autosomal dominant aniridia and congenital progressive cataract in three successive generations was recruited in the Eye Center of Second Affiliated Hospital, Medical College of Zhejiang University, Hangzhou, China. The study was performed in accordance with the Declaration of Helsinki and approved by the Zhejiang Institutional Review Board, and informed consent was obtained from all participants. The diagnosis was confirmed by ophthalmologic examinations, including visual acuity, slit-lamp examination, tonometer, keratometry, corneal topography, optical coherence tomography, ultrasonic A/B scan visual acuity, slit-lamp examination, corneal topography, optical coherence tomography, or a history of cataract extraction. Ocular photographs were taken by slit-lamp photography without pupil dilation. Twelve individuals (6 affected and 6 unaffected) from the family participated in the study (Figure 1). One hundred unrelated subjects were recruited as controls.

### Genomic DNA preparation and molecular analysis

Blood specimens (5 ml) from all the family members were collected in EDTA. Genomic DNA was isolated as previously described [12]. Briefly, 200 μl of the blood was incubated at 56 °C for 1 h in 200 μl of lysis buffer (provided in the kit) containing 25 ml of proteinase K. The purification procedure was carried out with QIAmp spin columns; the DNA was adsorbed onto the QIAmp silica membrane during a brief centrifugation step, washed twice, and eluted with 200 μl of distilled water. Genomic DNA samples from all the members of the family were screened for *PAX6* gene mutation by direct sequencing. All exons and flanking regions of *PAX6* were amplified by a polymerase chain reaction (PCR) using previously published primer sequences (Table 1) [13-15]. Briefly, PCR amplification conditions were: Reaction Mixture Set Up (25 μl); 50 ng of genomic DNA, 10× PCR buffer, 1.5 mM MgCl_2_, 0.2 mM dNTPs, 5 µmol each of sense and antisense primers and 2.5U of Taq DNA polymerase (Sangon Biotech, Shanghai, China). The cycling conditions for PCR were an initial denaturation step at 95 °C for 5 min, 10 cycles of touchdown PCR with 1 °C down per cycle from 60 °C to 50 °C, followed by 25 cycles with denaturation at 95 °C for 25 s, annealing at 55 °C for 25 s and extension at 72 °C for 40s, then finally extension at 72 °C for 10 min. PCR products were isolated by electrophoresis on 2% agarose gels and sequenced using the BigDye Terminator Cycle sequencing kit V 3.1 (ABI Applied Biosystems; Sangon Co., Shanghai, China) on an ABI PRISM 3730 Sequence Analyzer (ABI), according to the manufacturer’s instructions.

**Table 1:** PCR primers and product sizes of the *PAX6* gene.

**Exon**	**Sense primer (5′-3′)**	**Antisense primer (5′-3′)**	**Product size (bp)**
1 and 2	GCATGTTGCGGAGTGATTAG	CTCCTGCGTGGAAACTTCTC	645
3 and 4	GGACTTAGGGTTTGATGACAG	CCAGAAAGACCAGAGGCAC	644
5	TGAGGATGCATTGTGGTTGT	GAAATGAAGAGAGGGCGTTG	373
6	CGTAAGCTTGTCATTGTTTAATGC	AGAGAGGGTGGGAGGAGGTA	388
7	GGTTGTGGGTGAGCTGAGAT	AAGCCCTGAGAGGAAATGGT	333
8	GGCTGTCGGGATATAATGCT	CAAAGGGCCCTGGCTAAAT	355
9	AGGTGGGAACCAGTTTGATG	TGGGACAGGTTAGCACTGTGT	385
10 and 11	AGCAGTGGAGGTGCCAAG	TCTCAAGGGTGCAGACACAG	537
12	CAGACTTGTTGGCAGAGTTCC	TAAACACGCCCTCCCATAAG	345
13	CATGTCTGTTTCTCAAAGGGA	TTGTGTCCCCATAGTCACTGA	210

### Computational algorithms

Protein sequences among 8 different species were aligned using ClusatalW. The three diamensional structure of PAX6 paired domain was analyzed using the PyMOL tool.

## Results

### Clinical evaluation

**Figure 2:**
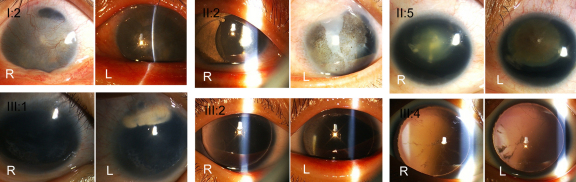
Slip lamp photographs of all the affected individuals. All the affected patients are had complete aniridia. The phenotypes are described and summarized in Table 2.

We identified a three-generation family with autosomal dominant aniridia and congenital progressive cataract (Figure 2). According to classification of aniridia phenotypes [16], all the six affected patients were categorized into iris 6 (complete aniridia). The best corrected visual acuity ranged from LP to 0.3. All of the affected patients were had horizontal nystagmus except I:2. Corneal curvature ranged from 35.2 to 43.0 (38.2±2.0 D in the minimal meridian and 40.8±2.2 D in the maximal meridian), and central corneal thickness (CCT) measured in the clear corneas (II:2, II:5, III:2 and III:4) ranged from 617 μm to 682 μm (642.5±24.4 μm). The above data demonstrated flattened and thickened corneas in the affected patients. Ectopia lentis was detected in patient II:4, but not in patient III:2 or III:4. There was no family history of other systemic abnormalities. All the clinical findings were summarized in Table 2.

**Table 2:** The clinical features of aniridia patients in the Chinese family.

**Patient**	**I:2**	**II:2**	**II:4**	**III:1**	**III:2**	**III:4**
Gender	F	F	M	M	F	F
Age (years)	63	43	38	20	11	11
BCVA R/L	LP/HM	0.25/HM	0.06/0.02	LP/LP	0.3/0.2	0.02/0.06
Nystagmus	NO	YES	YES	YES	YES	YES
Ptosis	YES	YES	NO	NO	NO	NO
Strabismus	Exotropia	Exotropia	NO	Exotropia	NO	Exotropia
Glaucoma	NA	YES	YES	YES	NO	NO
Corneal pannus	YES	NO	NO	YES	NO	NO
CCT R/L (μm)	NA	617/628	622/628	NA	671/682	634/658
Corneal curvature (D)	Min:37.5 Max:38.4	Min:35.2 Max:42.5	Min:39.9 Max:41.5	NA	Min:38.0 Max:38.4	Min:40.2 Max:43.0
CV	YES	NO	NO	YES	NO	NO
ECD (/mm3)	NA	1798	3333	NA	NA	NA
ACD R (mm)	2.37	3.25	2.07	NA	NA	2.14
Lens	Total Cataract	IOL	Nuclear cataracts	Aphakia	PSC	PSC
EL	NA	NA	YES	NA	NO	NO
Iris	Complete aniridia	Complete aniridia	Complete aniridia	Complete aniridia	Complete aniridia	Complete aniridia
C/D R/L	NA	0.8/0.4	NA	0.9/0.9	0.3/0.3	0.5/0.5
MT R/L (μm)	NA	216	NA	NA	NA	NA
AL R/L (mm)	25.0/26.6	NA	NA	25.4/25.0	21.4/21.2	19.7/19.7
CNS defect	NO	NO	NO	NO	NO	NO

Abbreviations: F, Female; M, Male; BCVA, Best-Correct Visual Acuity; R, Right eye; L, Left eye; LP, Light Perception; HM, Hand Motion; NA, Not Available; CCT, Central Corneal Thickness; CV, Corneal Vascularization; ECD, Endothelial Cell Density; ACD, Anterior Chamber Depth; IOL, Intraocular Lens; PSC, Posterior Subcapsular Cataracts; EL, Ectopia Lentis; C/D, Cup-to-disk Ratio; MT, Macular Thickness; AL, Axial Length; CNS, Central Nervous System.

### Genetic analysis

**Figure 3:**
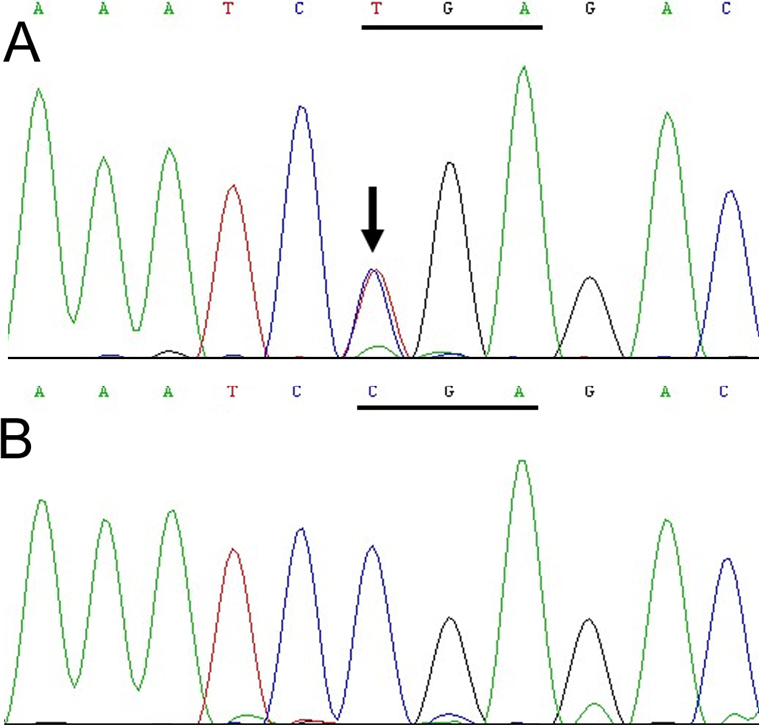
Sequencing results of the *PAX6* gene. **A**: The sequence of an affected member (individual II:4) is shown. **B**: The sequence of an unaffected member (II:3) is shown. A heterozygous mutation (c.307C>T) is detected in the exon 6 of *PAX6*.

By direct sequencing of the coding and flanking regions of *PAX6*, a heterozygous mutation (c.307C>T) was detected in all the six affected individuals (Figure 3). The mutation resulted in the substitution of an arginine codon for a termination codon (p.R103X). The c.307C>T mutation was detected neither in the unaffected members of the family, nor in any of the 200 control chromosomes that were analyzed from the same ethnic background.

### Computational analysis

**Figure 4:**
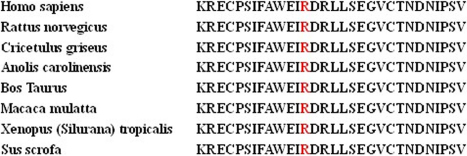
Multiple-sequence alignment in PAX6 from 8 different species. It demonstrates that R103 is highly conserved (highlighted in red).

**Figure 5:**
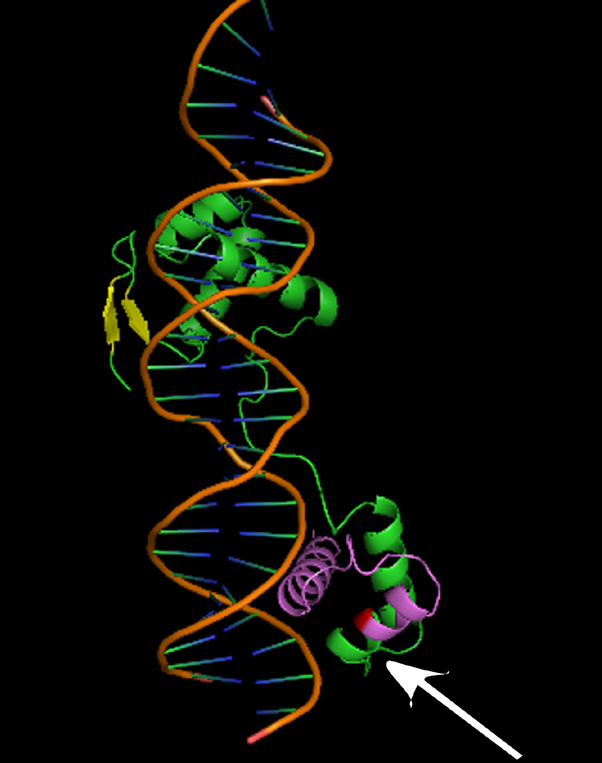
Stuctural modeling of the PAX6 paired domain. Cylinders and arrows represent α helices and β strands, respectively. The orange double helix in the center denotes DNA. The red segment represents the mutation point (R103). The structural model by PyMOL analysis shows the relationship between PAX6 paired domain and DNA.

The sequence alignment analysis showed that the Arg103 of human PAX6 protein (*Homo sapiens*, NP_000271.1) were highly conserved in various species including *Rattus norvegicus* (NP_037133.1), *Bos Taurus* (NP_001035735.1), *Macaca mulatta* (XP_001085332.1), *Xenopus (Silurana) tropicalis* (NP_001006763.1), *Sus scrofa* (NP_001231102.1), *Cricetulus griseus* (XP_003497614.1), and *Anolis carolinensis* (XP_003214735.1; Figure 4). PyMOL analysis showed that the p.R103X mutation was located at the DNA-binding domains of PAX6 (Figure 5) [17].

## Discussion

In the present study, we identified a recurrent mutation (c.307C>T) in *PAX6* in a three-generation aniridia and congenital progressive cataract family of Chinese origin. Although the c.307C>T mutation had been previously reported, it was associated with isolated aniridia or anophthalmia when the patient had compound mutations, and belonged to Caucasian population [18-20]. Our study first identified of the *PAX6* c.307C>T mutation in a large pedigree with aniridia and congenital progressive cataract. In this family, a thickened CCT (642.5±24.4 μm) was detected as a common clinical manifestation, which was consistent to the previous studies (631.6±50.8 μm) [21], compared to the measurement in healthy normal subjects (550.3±31.1 μm) [ 22]. AAK including central cornea thickness, flat cornea, corneal panus, and corneal vascularization indicated that *PAX6* had a crucial role not only in iris but also in corneal development. Broad phenotypic variability was observed in nystagmus, ptosis, strabismus, glaucoma, corneal pannus, corneal curvature, corneal vascularization, cataract subtype, ectopia lentis, axial length, and optic disc anomalies, showing phenotyically heterogeneous manifestations of the *PAX6* c.307C>T mutation.

Human PAX6 is composed of two DNA-binding domains: the paired domain (PD) of 128 amino acids and the homeodomain (HD) of 61 amino acids separated by a linker region of 79 amino acids, and is followed by a proline, serine, threonine-rich (PST) domain of 79 amino acids that have transcriptional trans-activation function [22]. It is a highly conserved transcription factor which regulates the tissue-speciﬁc expression of various molecules, hormones, and structural proteins. It is required for the development of the nervous system, eyes, nose, pancreas, and pituitary gland [23-26]. The sequence alignment analysis shows that Arg103 is highly conserved in vertebrates. Thus the c.307C>T mutation affecting the PD is inclined to be pathogenic and lead to congenital anomalies in eye development.

Nonsense-mediated decay (NMD) is the process by which mRNAs containing pre-mature termination codons (PTCs) are degraded before production of supposed truncated proteins [27,28]. It is reported that if a PTC is located 50–55 nucleotides 5′ to the last exon-exon junction, it is considered premature, and the mRNA is targeted for rapid decay [29]. Therefore, the *PAX6* c.307C>T mutation, occurring in exon 6 further upstream the last exon-exon junction, is supposed to result in NMD instead of truncated protein. Therefore, it probably behaves as loss-of-function mutants as predicted by haploinsufficiency [30]. Further investigation is still required to elucidate why the same non-sense mutation results in variation of phenotypes.

In conclusion, we detect a *PAX6* c.307C>T mutation in an aniridia and congenital progressive cataract family, and summarize the variable phenotypes among the patients. Our findings expand the phenotypic spectrum of aniridia in a different ethnic background.
